# Dietary patterns and inflammatory cytokine levels in healthy adult and adolescent women, whether pregnant or not: a prospective cohort study

**DOI:** 10.1590/1806-9282.20240538

**Published:** 2024-11-11

**Authors:** Vanessa Migray Moreto, Cristina Aparecida Falbo Guazzelli, Erika Ono, Karen Priscilla Tezotto Pendeloski, Edward Araujo, Silvia Daher

**Affiliations:** 1 Universidade Federal de São Paulo, Escola Paulista de Medicina, Department of Obstetrics – São Paulo (SP), Brazil.

**Keywords:** Dietary, Women, Pregnancy, Adolescent, Cytokines

## Abstract

**OBJECTIVE:**

The aim of this study was to assess the dietary pattern of healthy adult and adolescent women, pregnant and non-pregnant, and relate this profile to clinical and laboratory characteristics.

**METHODS:**

A prospective cohort study was carried out with 40 women who met the selection criteria: 10 non-pregnant adults, 10 pregnant adults, 10 non-pregnant adolescents, and 10 pregnant adolescents. Dietary data were collected using a registration form, a 24-h recall, and a food frequency questionnaire. Serum levels of interleukin 6 and tumor necrosis factor α were determined by capture ELISA.

**RESULTS:**

The majority of women were married (22.5%), had completed high school (57.5%), and were white (47.5%). Overall, only one (10%) pregnant adult reported smoking. Dietary supplement use was reported by eight (80%) pregnant adults, four (40%) pregnant adolescents, two (20%) non-pregnant adolescents, and no non-pregnant adults. Pregnant adolescents had a higher intake of omega-3 when compared to pregnant adults and non-pregnant adults (p=0.01 and 0.02, respectively). Pregnant adolescents consumed less minimally processed foods than pregnant adults, non-pregnant adults, and non-pregnant adolescents (p=0.008, 0.019, and 0.024, respectively). Serum levels of tumor necrosis factor α and interleukin 6 did not show statistical differences among the four groups (p=0.229 and 0.440, respectively).

**CONCLUSIONS:**

The dietary patterns of healthy adult and adolescent women, whether pregnant or not, were similar, with pregnant adolescents having a higher intake of omega-3. Pregnant adolescents ate less in natura (minimally processed) food than all the other women.

## INTRODUCTION

During pregnancy, the maternal organism develops physiological, anatomical, and immunological adaptations that are essential for the maternal metabolic needs and for the development and survival of the fetus^1^. Cytokines, proteins produced by different cells that can have an inflammatory or anti-inflammatory pattern, play a central role in this scenario. It is known that the cytokine profile is variable throughout pregnancy, and its observed effects can be beneficial or not, depending on the concentration, dose, and period evaluated^2^.

Science has demonstrated the influence of nutrition and dietary patterns on immunity and, in particular, on the induction of inflammation. The composition of commonly ­consumed foods gives the diet a pro- or anti-inflammatory character^3^. Unhealthy dietary patterns are associated with subclinical chronic inflammation and a higher incidence of chronic ­noncommunicable diseases and metabolic syndrome^4^.

The recurring search for convenience and low cost has led the population to increase their consumption of industrialized and ultra-processed foods instead of more natural ones. This transition is worrisome because we know that a diet rich in processed and ultra-processed foods negatively impacts health and nutritional status^5^. This, in turn, has a direct impact on the outcome of pregnancy, both for the mother and the newborn^6^. The benefits of a diet based on more natural foods for the prevention and maintenance of health are also known^7^.

There is no single dietary pattern that is considered ideal for the general population, adolescents, or pregnant women. Ultra-processed foods are generally consumed quickly because they require little or no preparation, are industrially prepared formulations, and are associated with a higher risk of clinical complications as well as inadequate micronutrient intake^8^. Pregnant women and adolescents are two groups particularly vulnerable to the effects of inadequate nutrition^9^. It has been observed that the mother's diet can affect both the weight of the pregnant woman and that of the newborn^10^.

Based on the literature, which demonstrates a clear ­relationship between diet, inflammation, and gestational outcomes, we hypothesize that women with a dietary pattern ­characterized by high consumption of ultra-processed foods will have higher levels of inflammatory cytokines and a higher risk of ­obstetric complications when compared to those with a diet rich in minimally processed foods.

The objective of this study was to assess the dietary ­pattern of healthy adult and adolescent women, pregnant and non-pregnant, and relate this profile to clinical and ­laboratory characteristics.

## METHODS

A prospective cohort study was carried out, and the participants were recruited from the Physiological Prenatal, Adolescent Prenatal, and Family Planning Outpatient Clinic at the Paulista School of Medicine—Federal University of São Paulo (EPM-UNIFESP). This study was approved by the Ethics and Research Committee of UNIFESP, on 03/06/2020, CAAE number: 38241620.4.0000.5505, and all participants signed the consent form.

The inclusion criteria adopted were:

1. pregnant women—singleton pregnancy with a live fetus and gestational age in the third trimester (28–32 weeks);
2. adolescents (pregnant or non-pregnant) between 10 and 19 years old;
3. adults (pregnant or non-pregnant) between 20 and 42 years old, with a pre-gestational body mass index (BMI) appropriate for their age according to the World Health Organization (WHO)^11^.

The exclusion criteria were women taking corticosteroids, antibiotics, immunosuppressants, antihistamines, or anti-inflammatories, as well as those with chronic clinical complications, organ transplants, and pregnant women with hyperemesis gravidarum.

The sample was for convenience since there are no similar studies that serve as a model for calculating sample size. A total of 40 participants, who met the selection criteria, were selected: 10 non-pregnant adults (NPA), 10 pregnant adults (PA), 10 non-pregnant adolescents (NPAd), and 10 pregnant adolescents (PAd).

Data collection was carried out in three stages. In the first stage, dietary data were collected using a registration form, a 24-h recall (24HR), and a food frequency questionnaire (FFQ), at the end of which a peripheral blood sample was taken. In the second stage, which occurred within 7 days after the first contact, an interview was conducted in person or by telephone to collect a new 24HR equivalent to a weekend day. In the case of pregnant women, a third contact (third stage) was made to collect postpartum data, which occurred in person or by telephone within 15 days after delivery.

The 24HR tool consists of a guided interview in which the participant reports and quantifies all food and beverages consumed in the 24 h prior to the interview (the day before the interview)^12^. The 24HR dietary recall was administered using a standardized form with a photo album illustrating the size of food portions and kitchen utensils. Based on this information, the food consumption reported in self-made measures such as "spoon" and "cup" was converted into grams by the DietSmart® program version 2018 (https://www.dietsmart.com.br), and from this, it was possible to obtain data such as nutrients and calories consumed during the day. The nutrients calculated were carbohydrates, proteins, and lipids (saturated fat, monounsaturated fat, polyunsaturated fat, cholesterol), sodium, vitamins A, D, B6, and B12, folic acid, calcium, iron, zinc, magnesium, copper and selenium, and fiber.

We used the FFQ, which consists of a fixed list of foods for which the interviewee reports the usual frequency of consumption and the size of the usual portion over a period of time^13^. This tool allowed the assessment of dietary intake in the months prior to the interview. The abbreviated version of the ELSA-Brazil Food Frequency Questionnaire was used for non-pregnant women^14^. For pregnant women, a questionnaire validated for Brazilian pregnant women was used^15^.

The questionnaires were analyzed in two ways: The first was to analyze the nutrients consumed—the foods reported by the subjects were tabulated in an Excel spreadsheet 2010 (Microsoft Corp., Redmond, WA, USA) and converted (taking into account their frequency and portion size) into daily portion equivalents. In the second form, the foods in each questionnaire were categorized according to their degree of processing, following the guidelines presented in the Food Guide for the Brazilian Population (2014), supported by the NOVA classification^16^. All foods were divided into three categories according to the degree of food processing: group 1—in nature or minimally processed foods; group 2—processed foods; and group 3—ultra-processed foods.

In order to assess food consumption according to the degree of processing, the frequency of consumption of each food was converted into a numerical value, i.e., a consumption score, calculated according to the equation Sn=(1/365) [(a+b)/2], where a and b are the number of days that each frequency of consumption represents in a 1-year period, according to Fornes et al.^17^. For the statistical analysis, the score of each food group according to its degree of processing was taken into account. Dietary intakes, i.e., calories, fiber, vitamins, and minerals, were calculated by taking the average of the data obtained from the calculations of the two 24HR applied to the FFQ data. Peripheral blood of 5 mL was collected and transported to the laboratory and was immediately processed, aliquoted, and stored at −80°C for subsequent cytokine measurement. Serum levels of interleukin 6 (IL-6) and tumor necrosis factor α (TNF-α) were determined by capture ELISA using commercial kits (DuoSet®, DY210) (R&D Systems, Minnesota, MN, USA) according to the manufacturer's instructions.

Statistical analysis was performed using JASP 0.16 statistical software (University of Amsterdam), and p<0.05 was considered statistically significant. Skewness and kurtosis, Kolmogorov-Smirnov, and Shapiro-Wilk normality tests were used to assess the distribution of quantitative variables. Student's t-tests (parametric variables) or Mann-Whitney tests (nonparametric variables) were used to analyze continuous variables. For analysis of variance between three or more groups, the ANOVA or Kruskal-Wallis test was used. When these tests were significant (p<0.05), Tukey's post hoc test was performed.

## RESULTS

We selected 40 women, pregnant or non-pregnant, adolescent or adult, the majority of whom were married (22.5%), had completed high school (57.5%), and were white (47.5%). The mean age and standard deviation (SD) of age and BMI of adolescent and adult women were 17.65±1.69 and 26.90±5.91 years (p<0.001) and 20.98±2.73 and 22.53±2.32 kg/m2, respectively (p=0.120).

Overall, only one (10%) pregnant adult reported smoking. Regarding contraceptive use, six (60%) adults and three (30%) adolescents reported previous use among pregnant women, and four (40%) adults and three (30%) adolescents had used contraceptives among non-pregnant women. Dietary supplement use was reported by eight (80%) PAs, four (40%) PAds, two (20%) NPAds, and no NPAs.

Based on the FFQ and 24HR, Table 1 shows the nutritional characteristics of the groups, considering the four groups of participants (NPA, PA, NPAd, and PAd). We observed that PAds seem to have a higher intake of omega-3 when compared to the group of PA and NPA (p=0.01 and 0.02, respectively).

**Table 1:** General nutritional profile of the groups.

	PA (n=10)		PAd (n=10)		NPA (n=10)		NPAd (n=10)		p
	Mean	SD	Mean	SD	Mean	SD	Mean	SD	
Kcal	1657.14	347.73	1636.13	337.59	1532.15	374.73	1616.50	315.41	0.850
Fiber	15.13	3.75	14.09	6.40	19.18	12.64	15.60	4.77	0.490
Cholesterol	224.06	54.74	209.47	46.09	192.23	50.70	204.27	36.15	0.510
Protein	68.14	16.79	67.44	16.83	62.38	19.66	67.10	13.95	0.860
Lipid	52.68	13.05	73.71	52.96	53.83	14.37	59,42	18.69	0.370
Saturated	16.65	4.12	15.42	3.95	17.01	9.51	16.88	6.22	0.940
Monosaturated	13.30	3.05	14.10	3.88	13.65	5.44	14.93	6.31	0.940
Polysaturated	9.06	3.59	9.32	3.38	15.31	22.34	10.87	3.95	0.600
Cholesterol	223.33	60.45	205.68	49.99	208.10	97.02	236.94	127.84	0.850
Omega 3	0.13	0.11	0.56	0.64	0.09	0.06	0.23	0.22	0.01a*; 0.02c*
Omega 6	1.74	1.07	2.91	1.83	1.55	0.73	2.52	1.86	0.130
Vitamin A	434.65	256.82	191.15	53.18	308.54	186.81	349.90	340.51	0.106
Vitamin B6	1.16	0.29	1.44	0.81	1.05	0.71	1.29	0.57	0.550
Vitamin B8	7.27	4.80	23.41	17.39	10.39	10.79	11.87	8.89	0.152
Vitamin B9	137.97	71.95	133.18	68.35	134.88	76.67	123.26	33.99	0.960
Vitamin B12	3.78	2.53	2.96	1.75	5.31	10.52	3.06	2.85	0.770
Sodium	2104.55	728.78	2232.44	562.27	1899.58	457.57	2325.90	830.41	0.510
Calcium	708.70	367.78	591.61	293.62	605.22	368.46	520.00	201.28	0.610
Iron	10.82	3.14	12.30	3.58	8.99	2.33	9.59	2.27	0.070
Zinc	8.03	2.28	7.48	2.96	4.84	2.24	6.51	3.07	0.057
Magnesium	182.20	43.82	190.11	77.82	154.98	73.88	165.13	50.47	0.590
Copper	0.75	0.24	0.78	0.37	0.69	0.32	0.79	0.30	0.890
Selenium	33.14	16.41	28.47	10.09	29.00	13.76	32.12	8.24	0.790
Iodine	70.03	39.11	97.62	49.08	63.25	44.47	107.29	118.49	0.440

NPA: non-pregnant adults; PA: pregnant adults; NPAd: non-pregnant adolescents; PAd: pregnant adolescents; SD: standard deviation. Analysis performed with Kruskal-Wallis test;

* p<0.05; Tukey's pos hoc. a: PA; c: NPA.

Table 2 shows that PAd consumed less minimally processed foods than PAs, NPAs, and NPAd (p=0.008, 0.019, and 0.024, respectively).

**Table 2:** Comparison of the general dietary profile of the groups, according to the degree of food processing.

	PA (n=10)		PAd (n=10)		NPA (n=10)		NPAd (n=10)		p-value
	Mean	SD	Mean	SD	Mean	SD	Mean	SD	
Minimally processed total score	0.205	0.051	0.117	0.043	0.197	0.071	0.194	0.063	0.008a, 0.019d, 0.024e
Total score processed	0.187	0.034	0.161	0.039	0.169	0.081	0.190	0.061	0.518
Ultra-processed total score	0.161	0.059	0.245	0.156	0.153	0.063	0.139	0.039	0.089

NPA: non-pregnant adults; PA: pregnant adults; NPAd: non-pregnant adolescents; PAd: pregnant adolescents; SD: standard deviation. Analysis performed with Kruskal-Wallis test;

* p<0.05; Tukey's post hoc. a: PA vs. PAd, d: NPA vs. NPAd, e: PAd vs. NPAd.

**Figure 1:**
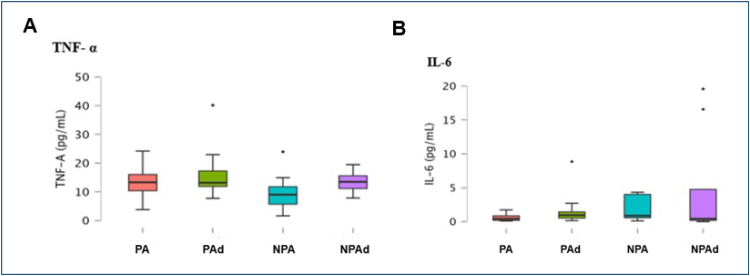
Comparison of cytokine profile ((A) tumor necrosis factor α and (B) interleukin 6) between groups presented as mean and standard deviation.

Regarding cytokines, TNF-α mean±SD for PA, PAd, NPA, and NPAd were, respectively, 12.66±5.88, 16.40±9.44, 9.76±6.31, and 13.56±4.20 pg/mL, without statistical ­difference between the groups (p=0.229). IL-6 mean±SD for PA, PAd, NPA, and NPAd were, respectively, 0.59±0.56, 1.77±2.60, 1.92±1.82, and 4.77±8.26, without statistical difference between the groups (p=0.440) (Figure 1). Although it was not significant, we noted a tendency in adolescents to present higher ­levels of inflammatory cytokines (TNF-A and IL-6).

## DISCUSSION

This study was proposed to start in 2021, being immediately affected and delayed by the COVID-19 pandemic and its extensive consequences, resulting mainly in a limited number of participants. With regard to diet, it is currently hypothesized that adolescents in general have a diet that is poor in nutrients and rich in potentially inflammatory foods, and that this deficiency may be related to adverse perinatal outcomes^18^. Our nutritional assessment showed little difference in the dietary profile of the participants. We would like to highlight the consumption of omega-3, which was significantly higher in PAds compared to PA and NPA. It is worth noting that this is a nutrient with a potential anti-inflammatory effect, which is important for maintaining good health^19^. The main sources of omega-3 are fish (salmon, sardines, tuna, etc.), seeds (chia, chestnuts, flaxseed), avocado, soy, and derivatives^20^. Studies have highlighted the importance of its intake throughout life, including during pregnancy, and there are indications that its consumption and supplementation can reduce the incidence of premature birth and is associated with a more appropriate newborn weight^21^. In addition, omega-3 is essential for the cognitive and visual development of the child before and after birth, and is essential during the last trimester of pregnancy and the first 2 years of the child's life^22^.

In order to identify any differences in the diets of the participating groups, we adopted the recommendations of the Brazilian Population Nutrition Guide of Brazilian Health Ministry and analyzed food consumption, taking into account the degree of food processing. This type of evaluation has been increasingly appreciated, and recent studies have shown a direct relationship between the consumption of ultra-processed foods and the occurrence of obstetric complications^23^.

As described in the literature, PAd consumed fewer healthy foods, i.e., minimally processed foods, than PA^24^. This finding was confirmed when the participants were subdivided into subgroups, with the pattern being present in both pregnant and non- PAd. However, this difference was not observed when the non-pregnant subgroups were compared with each other. It is possible that PAd eat a poorer quality diet because they are hungrier, more in a hurry to be satisfied, and choose foods that are quicker, more convenient, more palatable, and less expensive. It should also be noted that the most processed foods are the most accessible (cost and availability) and the most palatable. On the contrary, natural foods such as fruits, vegetables, and legumes require time to prepare and care to preserve, and because they are usually more expensive, they are rarely part of the basic food basket.

TNF-α and IL-6 are proinflammatory cytokines and are known to be involved in many pathological processes. Higher serum levels of TNF-α and IL-6 were observed in women with pregnancy-induced hypertension than normal pregnant women^25^. We observed higher serum levels of TNF-α in adolescents regardless of gestational status but without statistical significance. Similarly, there was a trend toward higher serum levels of IL-6 in adolescents compared to adults.

To bring more consistency to the results, it would be necessary to increase the number of inflammatory biomarkers, have access to more data weighted by an evolution curve throughout pregnancy, and, obviously, a larger sample size. However, this study can guide future investigations.

## CONCLUSION

The dietary patterns of healthy adult and adolescent women, whether pregnant or not, were similar. However, PAd had a higher intake of omega-3, a lower intake of natural (minimally processed) foods than all other women, and a tendency to have higher levels of inflammatory cytokines (IL-6 and TNF-α). These findings reinforce the urgent need for dietary interventions and the promotion of increased consumption of more natural foods.
